# Delayed Peri‐Implant Trochanteric Femoral Fractures After Intramedullary Nailing: Surgical Challenges in an Aging Society: A Two‐Case Report

**DOI:** 10.1155/cro/5170842

**Published:** 2026-04-10

**Authors:** Shunsuke Suzuki, Shunsuke Sato, Takeru Yokota, Takuya Kameda, Yasufumi Sekiguchi, Michiyuki Hakozaki, Yoshihiro Matsumoto

**Affiliations:** ^1^ Department of Orthopaedic Surgery, Fukushima Medical University School of Medicine, Fukushima City, Fukushima, Japan, fmu.ac.jp; ^2^ Higashi-Shirakawa Orthopaedic Academy, Fukushima Medical University School of Medicine, Fukushima City, Fukushima, Japan, fmu.ac.jp; ^3^ Department of Orthopaedic Surgery, Fujita Public General Hospital, Kunimi-machi, Fukushima, Japan

**Keywords:** case report, iatrogenic fracture, intramedullary nail removal, peri-implant trochanteric femoral fracture, super-aging society

## Abstract

**Introduction:**

Fractures, including peri‐implant femoral fractures, are increasing in aging populations. We present two trochanteric femoral fractures post–intramedullary nailing (IMN), highlighting preoperative planning for refracture risks and implant removal. These cases highlight potential surgical challenges in aging societies.

**Patient #1:**

The patient was a 91‐year‐old Japanese woman with a right trochanteric fracture, 10 years after IMN for a condylar open fracture. IMN removal was impossible; fixation used a dynamic hip screw. The fracture healed at 9‐month follow‐up.

**Patient #2:**

The patient was a 73‐year‐old Japanese man with a left trochanteric fracture, 14 years post‐IMN for a shaft fracture. An intraoperative fracture occurred during IMN removal, requiring later revision surgery.

**Discussion:**

In elderly patients, fracture fixation should consider future refracture risks and implant removal needs. Experiences from Japanese orthopedics in a super‐aged society offer insights for other aging populations.

## 1. Introduction

Hip fracture is common in elderly populations, and it significantly reduces an individual′s physical activity level [1]. Surgical intervention is generally required for a hip fracture [2], and the increase in the prevalence of hip fractures that has accompanied the aging of many societies worldwide has presented a critical issue that clinicians must address. In cases of peri‐implant femoral fractures, implant removal may be required before internal fixation [3]. However, the removal of an implant known as an intramedullary nail (IMN) has frequently been found to be difficult, potentially complicating the completion of the surgery [3]. The surgical treatment for a refracture that occurs after IMN fixation is thus often challenging.

However, implant removal is not mandatory in asymptomatic patients who are undergoing internal fixation procedures [4], and surgical planning that anticipates implant removal is not yet widely practiced; that is, when early stage elderly patients with longer life expectancy undergo IMN fixation, the possibility of subsequent refractures is not sufficiently considered. This issue is expected to become more evident in countries and regions that are experiencing advanced societal aging.

We describe the cases of two elderly patients with prior IMN fixation in the femur who experienced a later trochanteric fracture and for whom removal of the IMN was difficult or not possible. When planning IMN fixation in elderly patients with longer life expectancy, surgeons should anticipate the possibility of future peri‐implant fractures and prepare removal strategies or alternative constructs from the initial surgery. Their cases provide insights for orthopedic surgeons in countries that are facing societal aging.

## 2. Patient #1

### 2.1. History and Comorbidities/Medications

The patient was a 91‐year‐old Japanese woman. At the age of 81 years, she sustained a right femoral supracondylar open fracture due to a traffic accident. At another hospital, internal fixation was performed using an IMN (T2 Supracondylar Nail System, Stryker, Kalamazoo, Michigan, United States), and the fracture site was observed to have healed at 1 year postfixation. She was independently ambulatory without a cane, and she had no cognitive impairment.

Although a dual‐energy x‐ray absorptiometry (DXA) scan was not performed preoperatively, she had been prescribed a selective estrogen receptor modulator (SERM) following the previous fracture and had continued this medication for 10 years until the current injury.

### 2.2. Examination and Imaging

The patient was injured in an outdoor fall and was emergently transported to our facility due to difficulty in movement caused by right hip pain (Day 0). Plain radiographs and computed tomography (CT) examinations diagnosed a right femoral trochanteric fracture (AO Classification 31A2.2) (Figure 1). In Chan′s classification [5], this peri‐implant fracture was classified as N1A. She was admitted to our hospital on the same day for surgery planning. A preoperative DXA scan revealed a bone mineral density of 1.019 g/cm^2^ (T‐score −0.8, YAM 92%) at the lumbar spine (L1–L4) and 0.586 g/cm^2^ (T‐score −2.9, YAM 63%) at the proximal femur, establishing a diagnosis of osteoporosis.

Figure 1Patient #1, a 91‐year‐old woman. Preoperative plain radiographs and CT images at the time of injury. (a) Plain radiograph demonstrating an intramedullary nail (IMN) inserted and fixed from the distal aspect of the right femur, with evidence of bone union in the distal shaft. Cortical discontinuity was observed in the femoral trochanteric region, accompanied by a minimally displaced fracture. (b) Axial CT image revealing a complete fracture in the femoral trochanteric region. Bone tissue infiltration and adhesion within the interior of the tip of the IMN were observed. (c–f) Three‐dimensional (3D) reconstruction from CT images, revealing the four‐part fracture.

**Figure:**
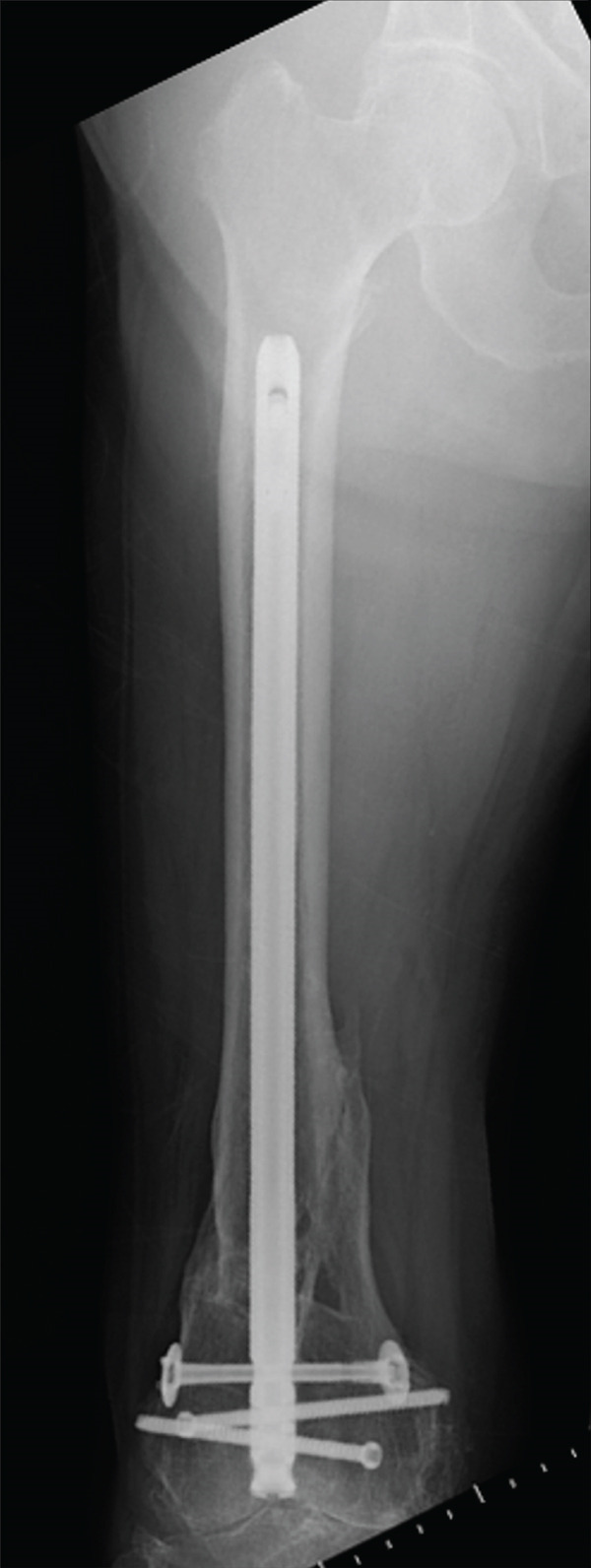
(a)

**Figure:**
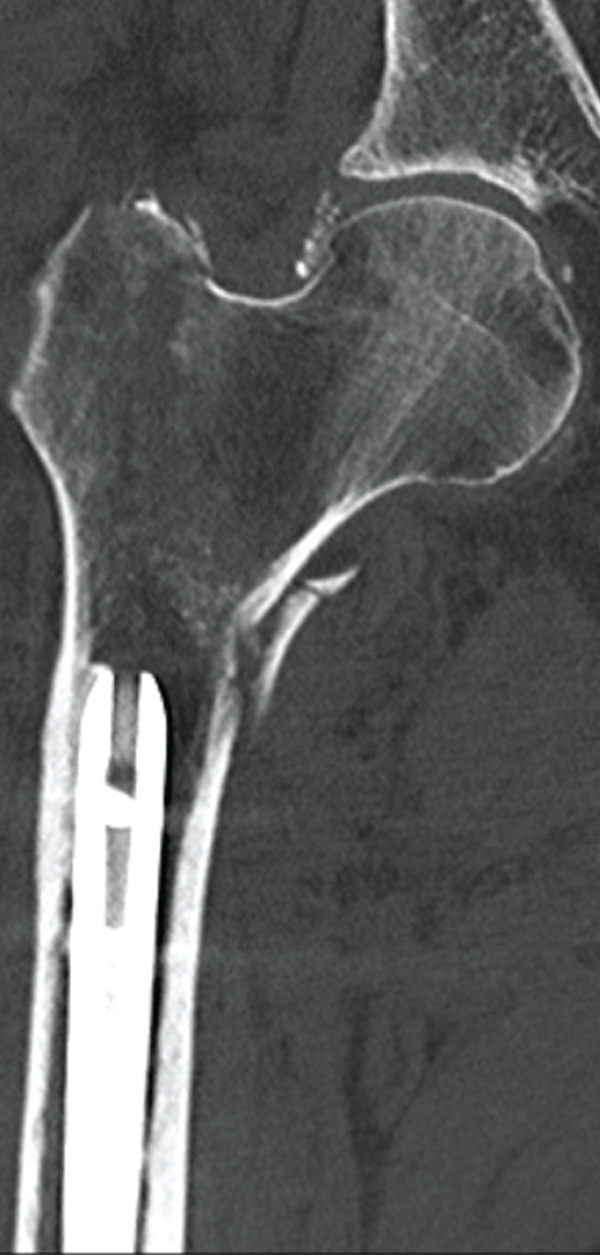
(b)

**Figure:**
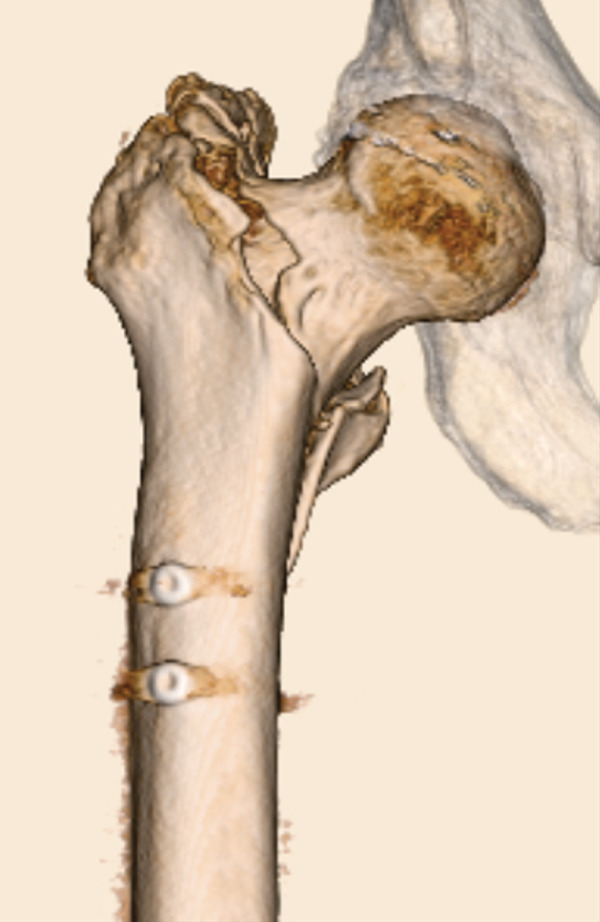
(c)

**Figure:**
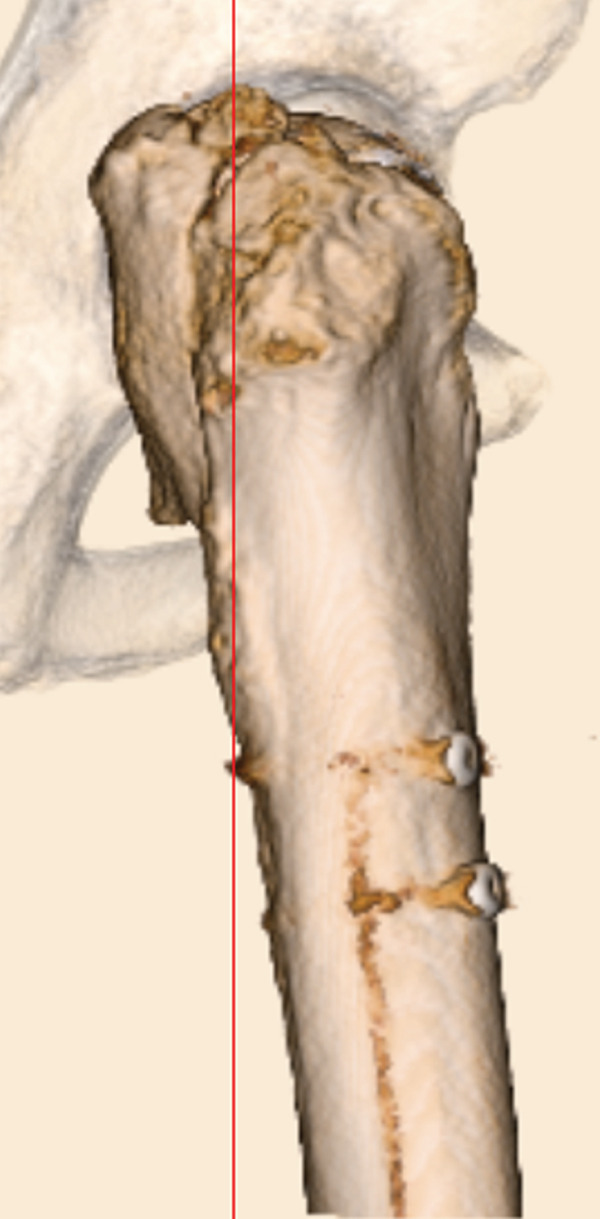
(d)

**Figure:**
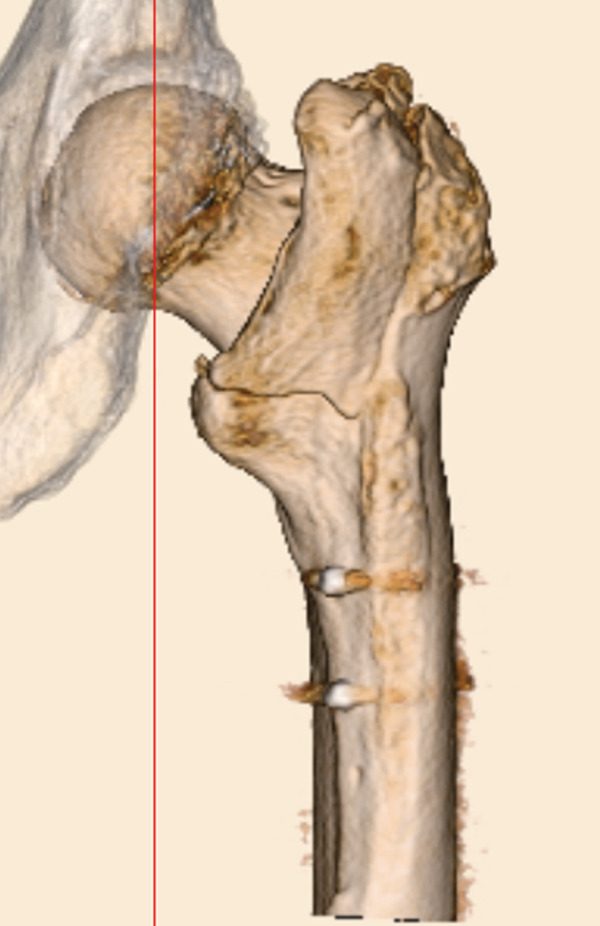
(e)

**Figure:**
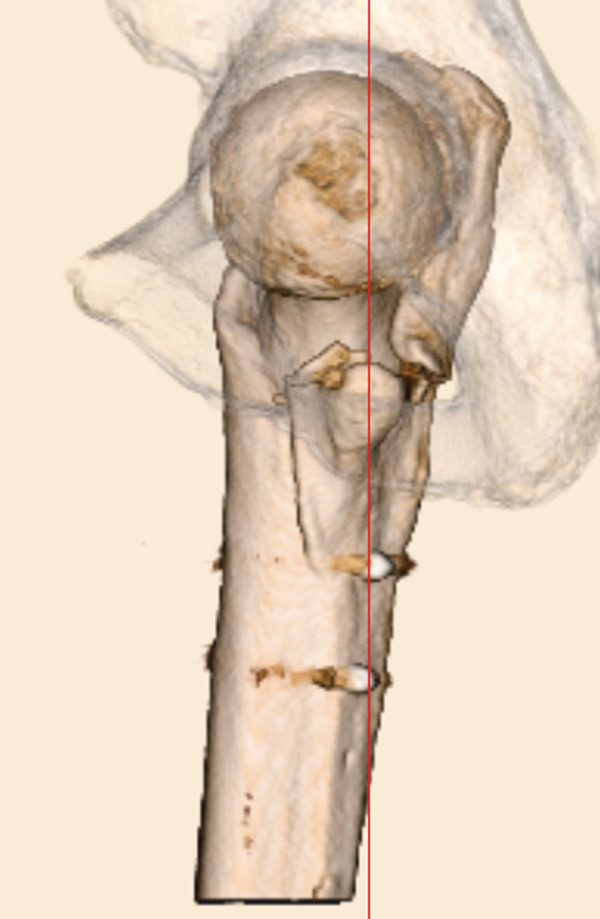
(f)

### 2.3. Operative Plan

The plan was to remove the IMN in the right femur and then perform long IMN fixation with antegrade proximal lag screw placement. As a second‐line option in case of difficult implant removal, internal fixation using a dynamic hip screw (LCP DHS Tube Plate System, DePuy Synthes, Raynham, Massachusetts) was also planned.

### 2.4. Intraop Findings/Complications

Six days after the injury, surgery was performed with the patient under general anesthesia on a traction table. Removal of the existing proximal locking screws was attempted first, but strong adhesion between the screws and bone tissue made removal difficult. During the attempt to extract the screws with a screwdriver, the screw heads became stripped and deformed, rendering removal of the screws impossible. Since other proximal screws could not be removed similarly, implant extraction was abandoned to avoid iatrogenic fracture.

Internal fixation was then performed, using the backup dynamic hip screw. Distal screws were used monocortically as positioning screws, and cable wiring was added for enhanced fixation (Figure 2a,b).

Figure 2Postoperative plain radiographs. (a, b) Anteroposterior and lateral views immediately after surgery. (c, d) Anteroposterior and lateral views at 49 days postoperatively. No displacement of the fracture site is evident, with reduced radiolucency at the fracture line, consistent with bone union.

**Figure:**
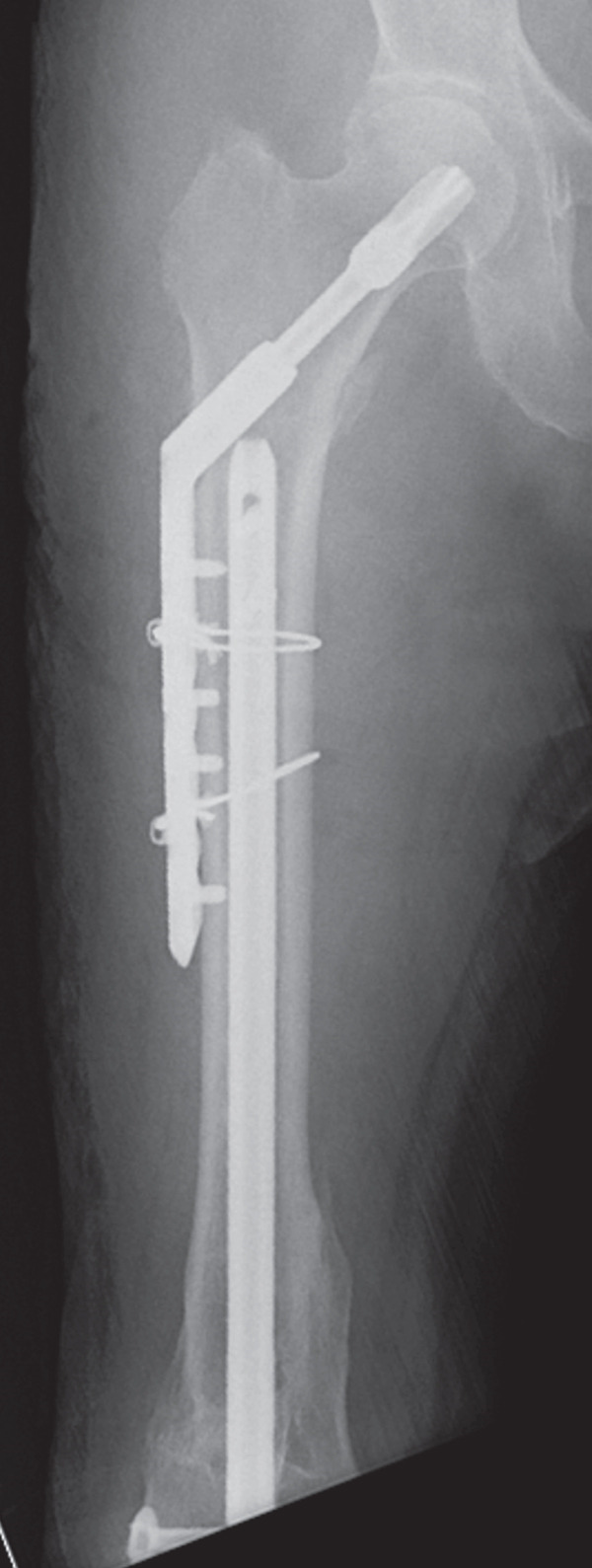
(a)

**Figure:**
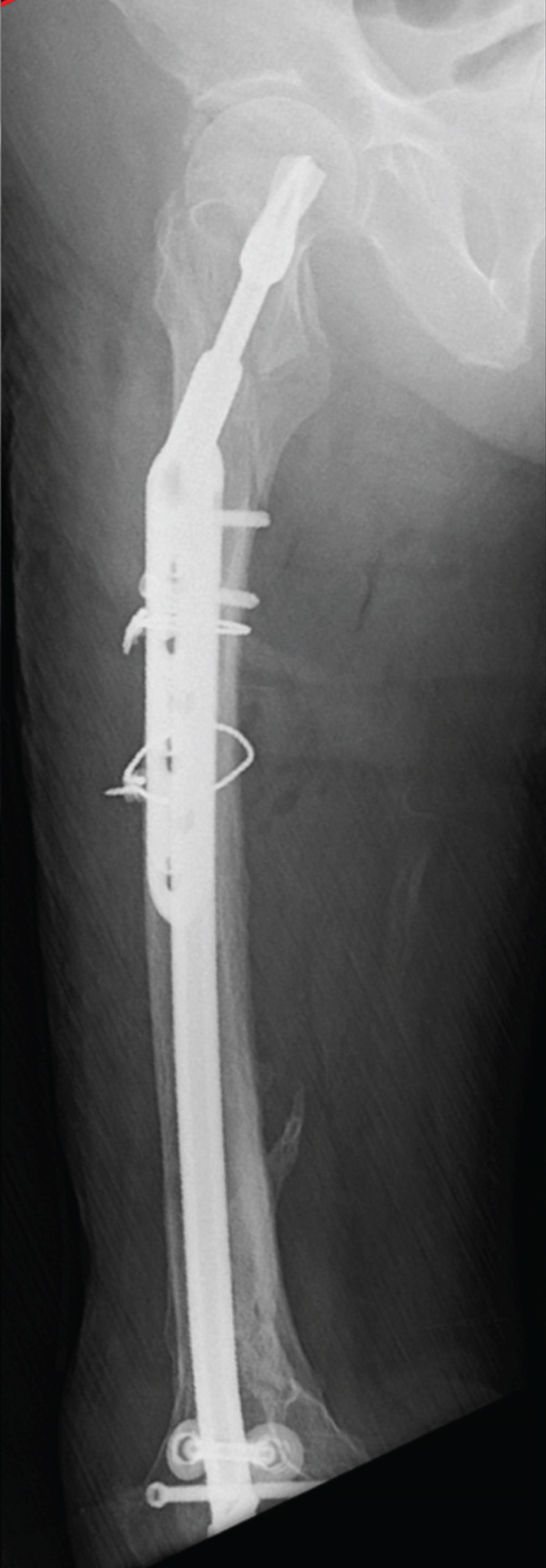
(b)

**Figure:**
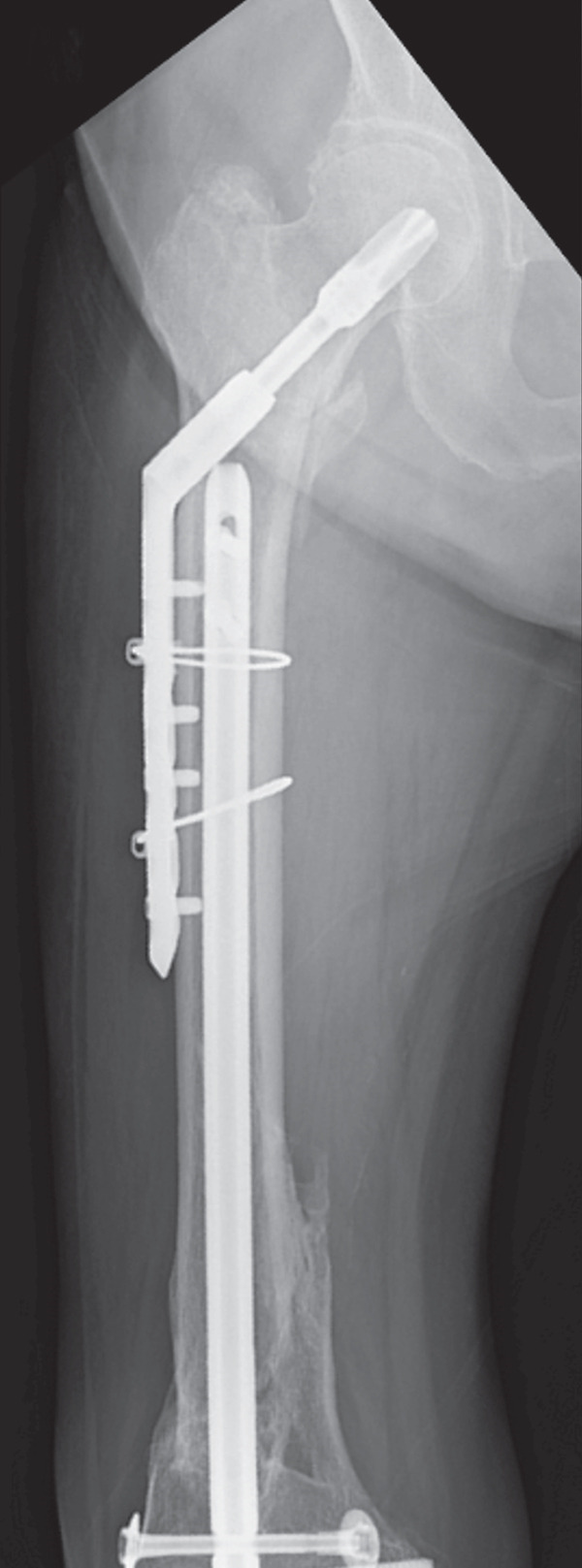
(c)

**Figure:**
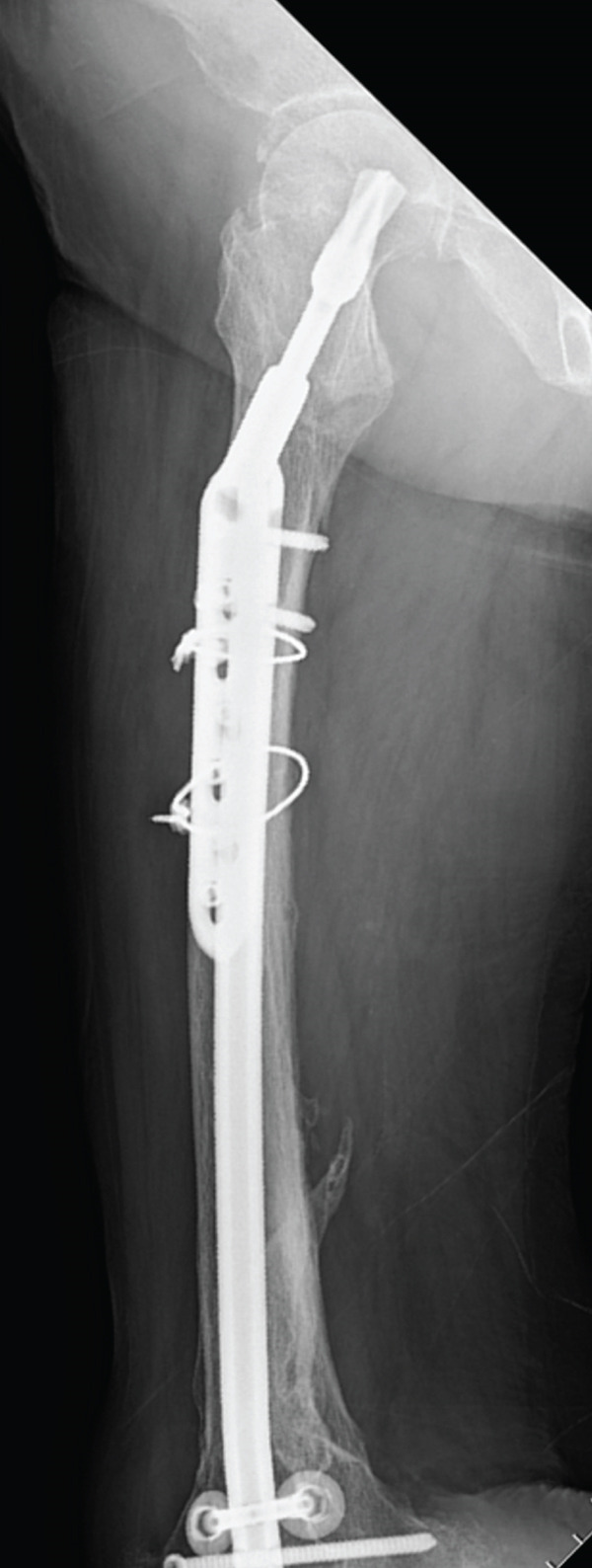
(d)

### 2.5. Postop Protocol

Joint range‐of‐motion training and full weight‐bearing were permitted from postoperative Day 1, as the DHS construct provided immediate stability without reliance on the old IMN. This early mobilization protocol was chosen because complete implant retention with supplementary fixation reduces the risk of prolonged nonweight‐bearing in frail elderly patients. At 9 months postoperatively, the antiosteoporosis medication was switched from a SERM to denosumab.

### 2.6. Outcome and Follow‐Up

Walker‐assisted gait training began on Day 18, and independent walking with a pickup walker was achieved by Day 25. Radiographic bone union was confirmed on Day 49 (Figure 2c,d). The patient was discharged home at 2 months postoperatively and was able to walk independently with a cane at the 9‐month final follow‐up.

## 3. Patient #2

### 3.1. History and Comorbidities/Medications

The patient was a 73‐year‐old Japanese man. At the age of 59 years, the patient had undergone IMN (ACE Femoral Nail System, ACE Medical Company, El Segundo, California, United States) fixation for the left femoral shaft fracture. The patient had no other notable medical history or medication records. No diagnosis of osteoporosis had been made, and no antiosteoporosis medications had been prescribed.

### 3.2. Examination and Imaging

The patient was injured in an outdoor fall. He was emergently transported to our facility with the chief complaint of difficulty in movement due to left hip pain. Plain radiographs and CT revealed a left femoral trochanteric fracture (AO Classification 31A1.2) (Figure 3). In Chan′s classification [5], this peri‐implant fracture was classified as N1A. A DXA scan was not performed preoperatively.

Figure 3Patient #2, a 73‐year‐old male. Preoperative plain radiographs and CT images at the time of injury. (a, b) Plain radiographs showing anterograde insertion of an IMN from the proximal left femur. A minimally displaced fracture extended from the proximal end of the IMN toward the lesser trochanter. (c) Axial CT image depicting the complete fracture in the femoral trochanteric region, with the fracture occurring at the site of the locking screw in the lesser trochanter area. (d) The 3D reconstruction from CT images, demonstrating a simple fracture.

**Figure:**
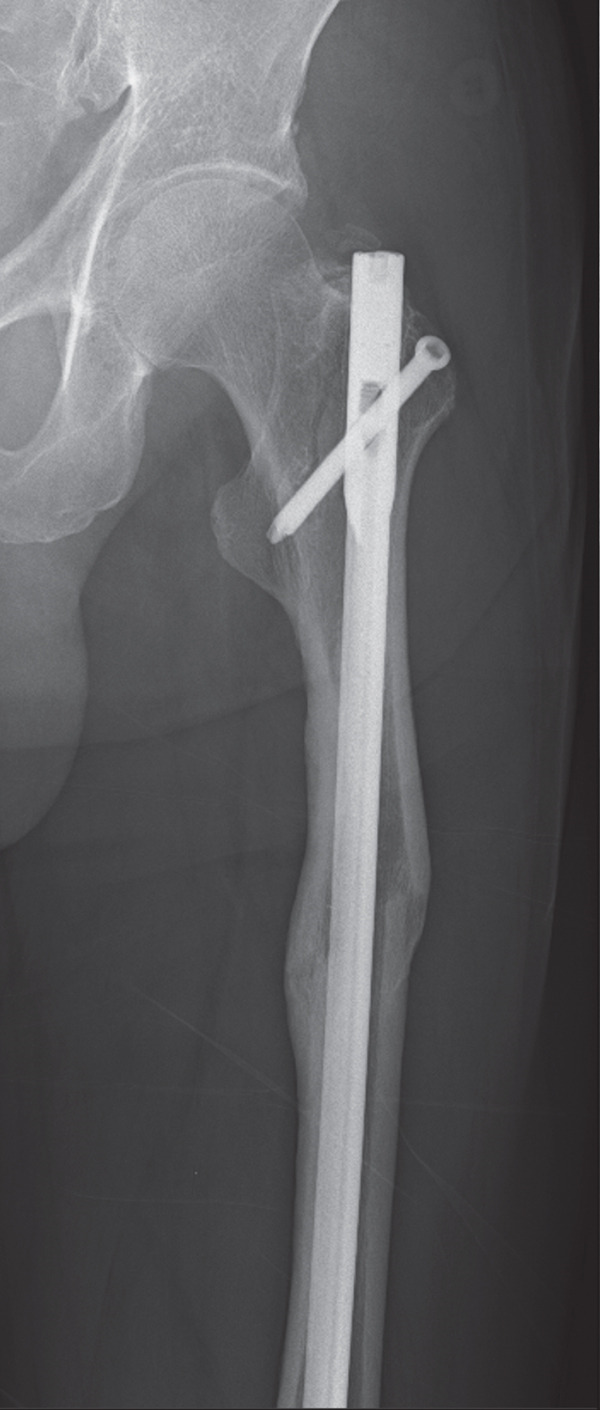
(a)

**Figure:**
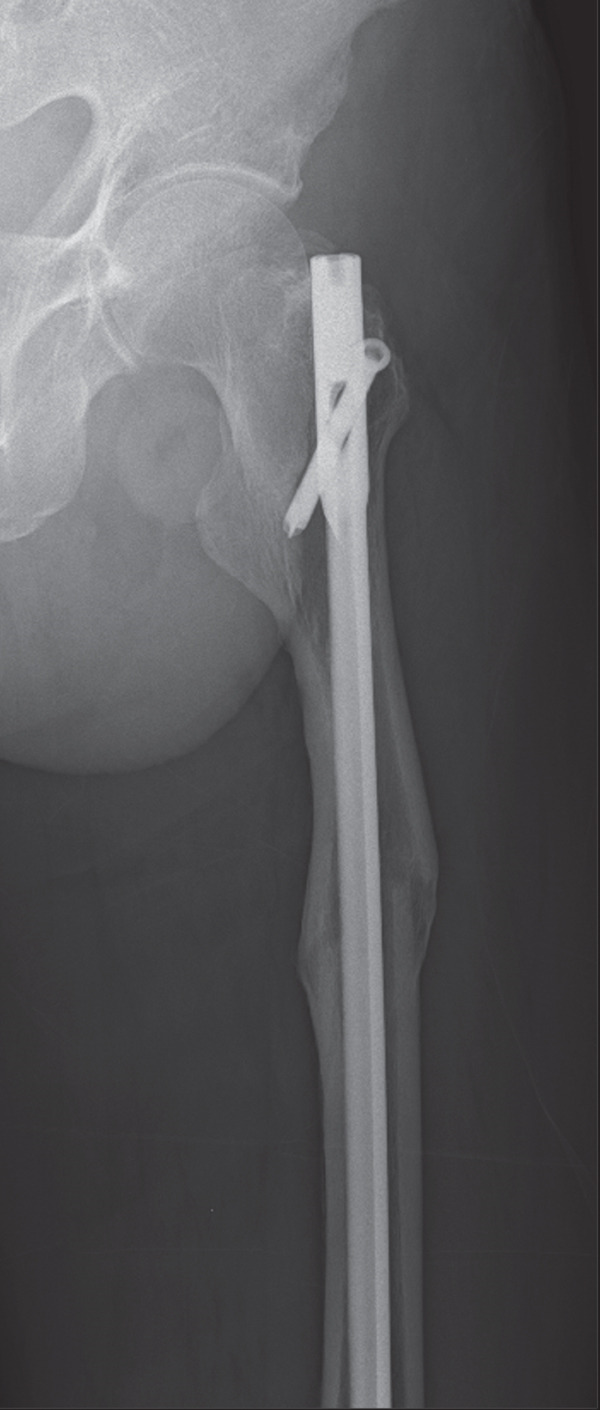
(b)

**Figure:**
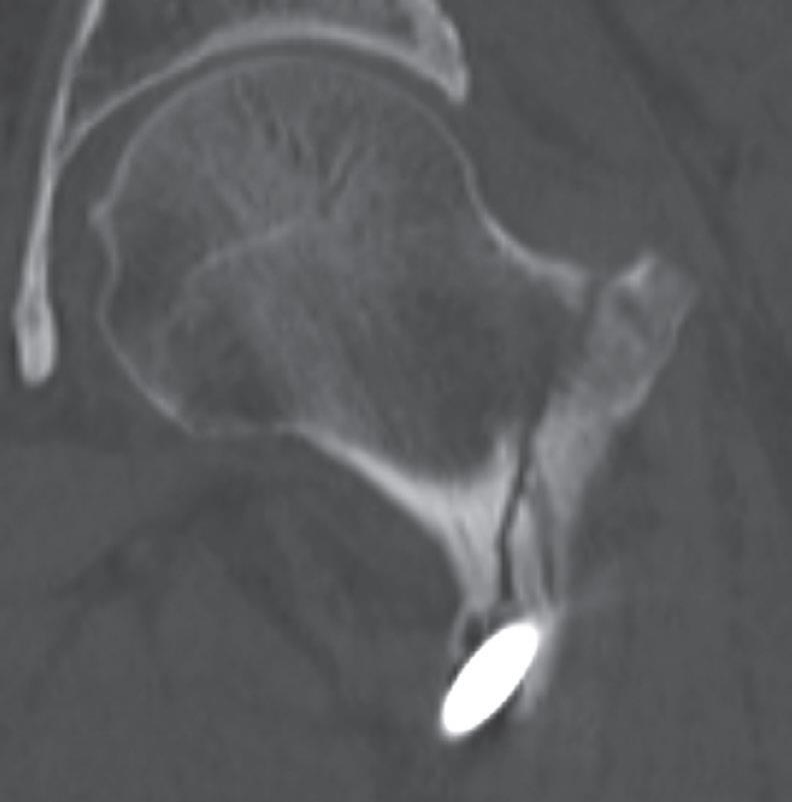
(c)

**Figure:**
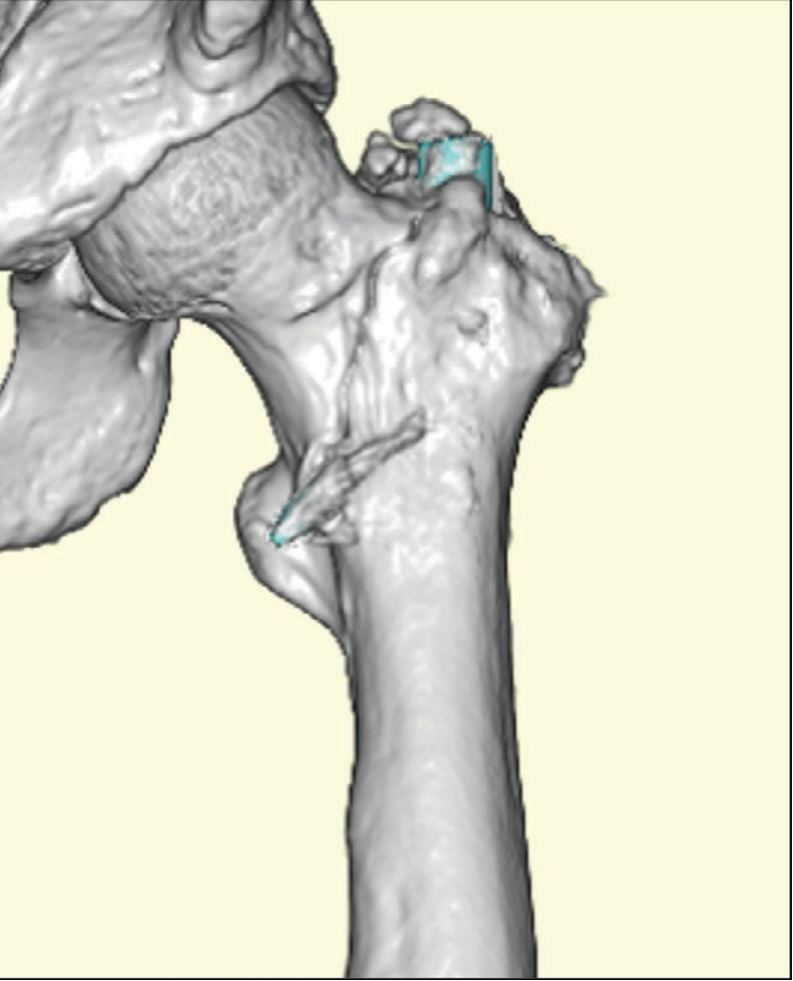
(d)

### 3.3. Operative Plan

The previously fixed IMN was an implant capable of proximal lag screw insertion. However, the preoperative planning revealed that the depth and rotational direction of the lag screw were not oriented toward the center of the femoral head. Internal fixation with antegrade IMN insertion after removal of the IMN was thus planned.

### 3.4. Intraop Findings/Complications (First Surgery)

The patient underwent the first surgery under general anesthesia on a traction table. Removal of the previously inserted IMN was attempted first using the Biomet ACE femoral nail extraction set (T065; Biomet, Warsaw, Indiana, United States). No problem was encountered regarding the removal of the end cap and locking screws. An extraction device was attached to the IMN, and its removal was attempted using the backstroke technique, but extraction became impossible after the nail advanced 3 cm. Repeated extraction attempts resulted in the breakage of the two extraction devices.

Next, bone tissue around the IMN at the narrowest part of the medullary canal was drilled with a surgical drill, but this did not enable the IMN′s removal. The distal femur was then windowed to release additional obstructing factors, and the removal of the IMN was successful; however, an iatrogenic femoral fracture occurred in the distal femoral shaft. Soft wire was manually looped around the intraoperative fracture site and secured by twisting, after which an antegrade long IMN (Gamma3 Nail System, Stryker) was inserted, with a lag screw placed in the femoral neck for fixation (Figure 4).

Figure 4Plain radiographs after the patient′s first surgery. (a, b) Anteroposterior and lateral views confirming satisfactory femoral alignment. (c) Lateral view of the distal femur, revealing the intraoperative fracture in the distal shaft. Distally to the fracture line, only two screws were present, with one positioned nearly at the fracture site, suggesting inadequate fixation. White arrows indicate the site of the iatrogenic distal shaft fracture; the star indicates inadequate distal screw placement near the fracture line.

**Figure:**
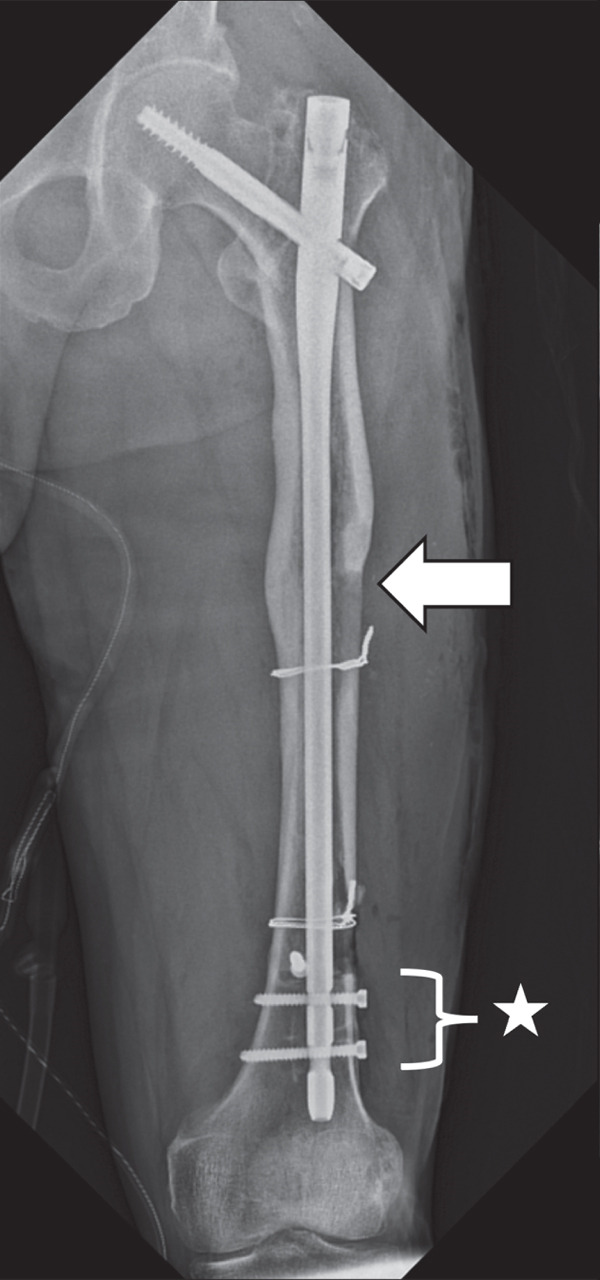
(a)

**Figure:**
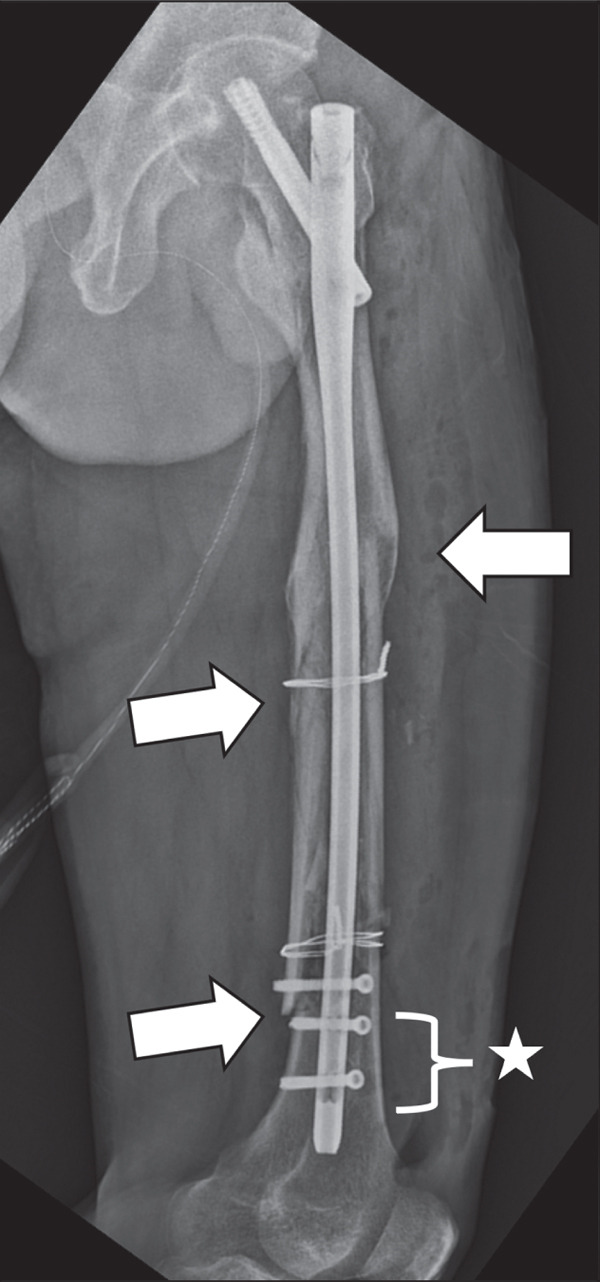
(b)

**Figure:**
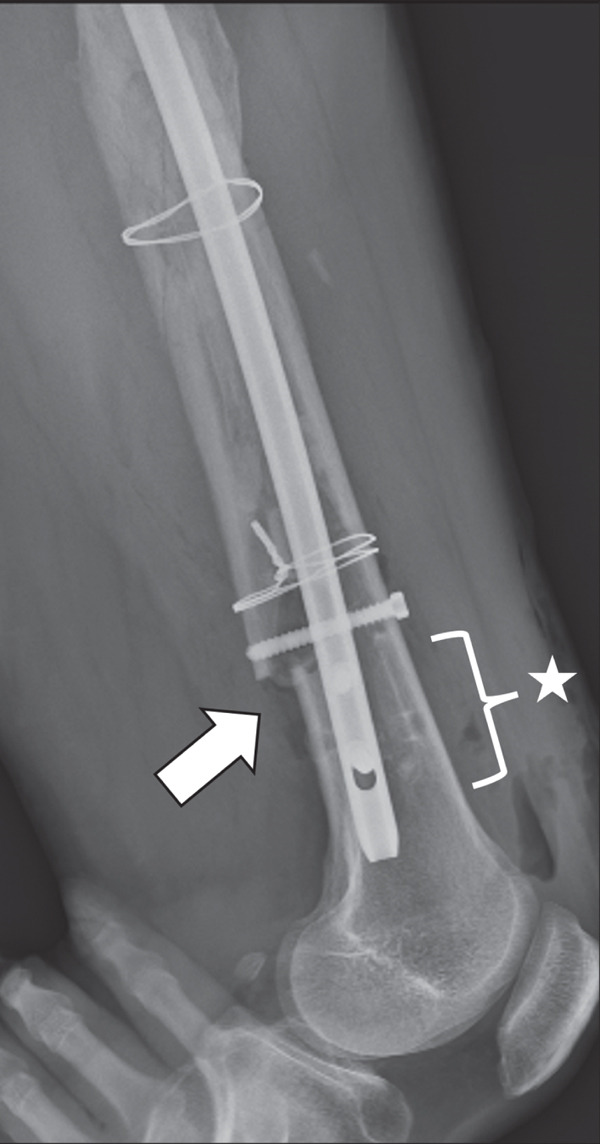
(c)

### 3.5. Intraop Findings/Complications (Second Surgery)

**Figure 5:**
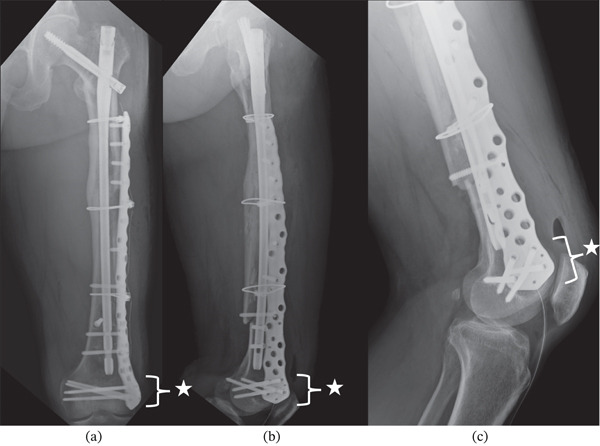
Plain radiographs taken after the patient′s second surgery. (a, b) Anteroposterior and lateral views, showing maintained femoral alignment from the first surgery, with additional plate fixation. (c) Multiple screws were fixed to the distal femur, providing sufficient stability to the femoral condylar fragment. Stars indicate additional stabilizing screws from the second‐stage periprosthetic plate.

Five days after the patient′s first surgery, additional fixation was performed because the initial long IMN construct provided inadequate distal stability for safe early mobilization. A periprosthetic plate had not been prepared as a backup during the index procedure; therefore, a staged revision with a plate (NCB Periprosthetic Femur Plate System, Zimmer Biomet, Warsaw, Indiana) and allogeneic iliac bone graft was required to achieve sufficient stability for rehabilitation (Figure 5).

### 3.6. Postop Protocol

Joint range‐of‐motion training began the day after the second surgery. Nonweight‐bearing was maintained until 8 weeks postoperatively, progressing to one‐third weight‐bearing at Week 9, one‐half weight‐bearing at Week 11, and full weight‐bearing at Week 14. This graduated protocol was chosen because the staged revision required time for biological incorporation of the bone graft and to minimize stress on the peri‐implant site.

Although neither preoperative nor postoperative DXA was performed, the low‐energy trochanteric femoral fracture in this 73‐year‐old patient was regarded as a major fragility fracture. Subcutaneous teriparatide (Teribone, Asahi Kasei Therapeutics Corporation, Tokyo, Japan) 28.2 *μ*g twice weekly was therefore initiated on postoperative Day 7.

### 3.7. Outcome and Follow‐up

Radiographic bone union was confirmed at 4 months postoperatively. At the 12‐month final follow‐up, the patient was able to walk independently without aids.

## 4. Discussion

We treated two patients with a femoral trochanteric fracture that occurred after IMN fixation. In both cases, IMN removal was difficult, complicating the surgical process. In elderly patients, the risk of refracture after IMN fixation should be considered. During the initial IMN surgery for an elderly patient, the possibility of future new fractures requiring implant removal should be taken into account.

Hip fracture is the most common fractures in elderly populations [1], and these fractures generally require surgical intervention [2]. Previously inserted IMNs have impacted surgical approaches differently, as is illustrated by the present patients′ cases. In Patient #1, osteosynthesis was feasible without implant removal, whereas in Patient #2, implant removal was necessary and resulted in an intraoperative fracture. These observations underscore the importance of taking potential future fractures and implant removal challenges into account when an initial IMN′s removal is being planned.

### 4.1. Peri‐Implant Femoral Fractures and Aging Societies

Global aging continues to progress, affecting entire medical systems worldwide, and the increase in fractures among the elderly is a particularly relevant problem in the field of orthopedics [6]. Japan, as one of the few “super‐aged” societies, can serve as a valuable model case for countries facing societal aging.

According to the World Health Organization (WHO) report on the progress of aging societies, the number of individuals aged ≥ 60 years is expected to reach one‐sixth of the total global population by 2030 [7], indicating that (i) life expectancy is extending worldwide and (ii) the proportion of the elderly in many countries and regions is increasing. Over 30% of Japan′s population is aged ≥ 60, which is unprecedented worldwide [7]. The average life expectancy of Japanese citizens in 2024 was 81.09 years for men and 87.13 years for women [8]. Japan is expected to experience even greater societal aging in the future. With a further extension of the average life expectancy, the period of high fracture risk will lengthen. Considering that the risk of hip fracture increases with age [6], the number of hardware‐related fractures that occur after an initial femoral surgery is also expected to increase.

### 4.2. Hardware Removal and Internal Fixation

Considering the present cases, IMNs with slit structures or those in which new bone has extensively adhered to the implant should prompt surgeons to anticipate removal difficulties from the initial planning stage.

The breakage of the extraction device in Patient #2 was not due to a problem with the extractor itself; rather, we believe it resulted from severe bone tissue adhesion combined with the nail. It is reported that this implant has a higher complication rate of iatrogenic fractures during removal [9], and the refracture rate in cases where actual removal was attempted was reported to be 21% [10]. It is recognized that new bone adhering to the slit portion causes extraction difficulties.

A variety of auxiliary techniques have been reported for such difficult extractions. In reviews of femoral IMN removal cases, prolonged operative times and removal difficulties in delayed cases and with titanium implants have been documented [11]. Kose et al. comprehensively organized various strategies for difficult removal and demonstrated the usefulness of auxiliary techniques such as overreaming and cortical windowing, in addition to standard extraction methods [12]. Arif et al. conducted a systematic review of case studies on the extraction of bent or broken tibial IMNs, analyzing the success rates and complications (including iatrogenic fractures) of standard extraction, hook extractors, medullary canal enlargement (overreaming), cortical windowing, and other auxiliary techniques in detail, concluding that early transition to alternative fixation should be considered in difficult cases [13].

In Patient #2, overreaming and cortical windowing were selected as auxiliary techniques based on the intraoperative findings. Although IMN removal was successful, it was accompanied by an iatrogenic fracture. This experience demonstrates that iatrogenic fracture during nail removal may sometimes be difficult to avoid. It underscores the importance of meticulous preoperative strategic planning, preparation of multiple backup instruments, and flexible intraoperative decision‐making—including early transition to alternative fixation methods.

### 4.3. Surgical Decision‐Making Algorithm for Peri‐Implant Trochanteric Femoral Fractures

Several classification systems have been reported for fractures occurring around bone fixation materials such as IMNs [5, 14–16]. Although these systems exist for peri‐implant fractures around bone fixation devices, they differ in various parameters such as implant type, fracture location, healing status of the original fracture, and fracture morphology. As a result, no single most effective classification has been established to date. However, all of these systems recognize the relative position between the implant and the fracture site as a particularly useful factor for classification.

Among them, the Chan classification [5] is the most widely cited. This system categorizes fractures according to implant type (N for nail), location relative to the implant (1 for at/around the proximal tip), and healing status of the original fracture (A for healed) and also proposes a treatment algorithm based on the classification. Based on this classification, both of our fractures were N1A. According to the algorithm, the general recommendation would be to remove the existing IMN and insert a longer nail with an extended fixation range, as we initially planned. However, in our cases, complete removal was difficult due to severe adhesion, making this approach challenging.

In peri‐implant trochanteric femoral fractures, morphological classification and the corresponding treatment algorithm are important. At the same time, we believe that a stepwise approach for practical surgical decision‐making that also considers the feasibility of implant removal is equally effective (Figure S1).

Clinicians should bear in mind that even when implant removal is judged necessary, there are situations in which removal must be attempted. In some cases, an iatrogenic fracture may be unavoidable. Nevertheless, it is equally important to formulate a meticulous surgical plan to avoid such complications. Surgeons should anticipate every possible scenario in advance, determine the surgical strategy accordingly, and prepare all necessary instruments and backup constructs before commencing the operation.

### 4.4. Considerations for Initial IMN Fixation

When the placement of an IMN in the femur is planned, two strategies should be considered. One possible strategy is to add a support mechanism, such as a lag screw directed toward the femoral neck, which may help prevent future trochanteric fractures. This approach is consistent with strategies used for atypical femoral fractures [17], as unprotected regions may be at risk of stress fractures [18]. Incorporating screws toward the femoral head can help protect the neck from subsequent fragility fractures.

The second strategy is to leave sufficient space for adding a proximal support mechanism if a trochanteric fracture develops later. In our Patient #1′s case, for example, dynamic hip screw fixation was possible without the major complications that are associated with IMN removal.

Given the increasing emphasis on secondary fracture prevention in elderly patients [19], surgical procedures that reduce the risk of future fractures should be prioritized. The first strategy mentioned above is suggested as a useful approach for most cases to prevent future trochanteric refractures. However, when the initial fixation method precludes proximal lag screw insertion—as in Patient #1′s first fracture—the second strategy represents a practical alternative.

In addition, it remains unclear how differences in implants that allow proximal lag screw insertion or augmentation of lag screws (multiple screws, cement, or artificial bone) contribute to fracture prevention. For example, dual‐screw integrated systems are superior to single‐screw devices in preventing cutout and providing rotational stability [20], but this is a separate issue from the risk of peri‐implant secondary fractures. The reason is that these fractures are primarily caused by stress concentration around the nail [21]. Whether any specific implant or lag screw fixation method is most effective for preventing refractures of the proximal femur under the proximal support strategy proposed here requires further study.

A potential concern with the second strategy is the potential occurrence of a femoral neck fracture after IMN fixation. Displaced neck fractures usually require a hemiarthroplasty or total hip arthroplasty [22], necessitating removal of the IMN and carrying a risk of intraoperative fracture. Difficulties during IMN removal are frequently reported [4], and orthopedic surgeons should therefore be familiar with techniques for managing such complications.

### 4.5. Limitation

Regarding secondary fracture prevention, although neither preoperative nor postoperative DXA measurements were performed in Patient #2, the trochanteric femoral fracture resulting from a ground‐level outdoor fall was treated as a major fragility fracture. Antiosteoporosis therapy with teriparatide was promptly initiated on postoperative Day 7 without BMD confirmation. Although this early intervention itself does not completely deviate from current best practices for reducing refracture risk in elderly patients following hip fractures, DXA measurement should have been performed for appropriate osteoporosis management.

## 5. Learning Points/Clinical Pearls

• In super‐aging societies, initial IMN fixation for femoral fractures in patients with long‐life expectancy should include surgical planning that anticipates the possibility of future peri‐implant fractures and implant removal.
• When IMN removal is difficult due to endosteal bone ongrowth, the risk of iatrogenic fracture should be considered, and transition to backup fixation should be contemplated.
• Surgical planning should not rely solely on conventional morphological classifications of peri‐implant fractures; instead, it should comprehensively incorporate information on the feasibility of implant removal.
• Orthopedic surgeons must make every possible preparation for nail extraction and be thoroughly familiar with strategies for managing complications during implant removal.

## 6. Conclusion

An increase in hardware‐related fractures is expected in countries with aging populations. Orthopedic surgeons treating femoral fractures in elderly patients should consider the potential for future hardware‐related fractures during surgical planning, at the timepoint of the initial fracture′s treatment.

## Author Contributions

Shunsuke Suzuki: investigation (Patient 2), writing—original draft (Patient 2); Shunsuke Sato: resources (Patient 2), writing—review and editing; Takeru Yokota: conceptualization, investigation, visualization, writing—original draft; Takuya Kameda: validation; Yasufumi Sekiguchi: resources (Patient 1), writing—review and editing; Michiyuki Hakozaki: writing—review and editing; Yoshihiro Matsumoto: writing—review and editing, supervision.

## Funding

No funding was received for this manuscript.

## Ethics Statement

This study was conducted in accordance with the ethical standards of the institutional and national research committees. Ethical approval was not required for this anonymized case report.

## Consent

Written informed consent for the publication of their case details and images was obtained from the patients.

## Conflicts of Interest

The authors declare no conflicts of interest.

## Supporting information


**Supporting Information** Additional supporting information can be found online in the Supporting Information section. Figure S1: Decision‐making algorithm for the surgical management of peri‐implant trochanteric femoral fractures after intramedullary nailing. The algorithm is divided into two main pathways depending on whether implant removal is deemed necessary. Pathway 1 (removal necessary) includes detailed subbranches for removal attempts and the risk of iatrogenic fracture. Pathway 2 (removal not necessary) shows options for supplementary fixation around the retained implant. Patient #1 was managed according to Pathway 2 (additional fixation without removal using a dynamic hip screw), while Patient #2 followed Pathway 1 with successful nail extraction despite an iatrogenic fracture.

## Data Availability

The data that support the findings of this study are available on request from the corresponding author. The data are not publicly available due to privacy or ethical restrictions.
