# Supplementary figures and images for: Delayed Peri‐Implant Trochanteric Femoral Fractures After Intramedullary Nailing: Surgical Challenges in an Aging Society: A Two‐Case Report
Source: Case Rep Orthop. 2026 Apr 10;2026:5170842. doi: 10.1155/cro/5170842 (PMC13069183; doi:10.1155/cro/5170842)

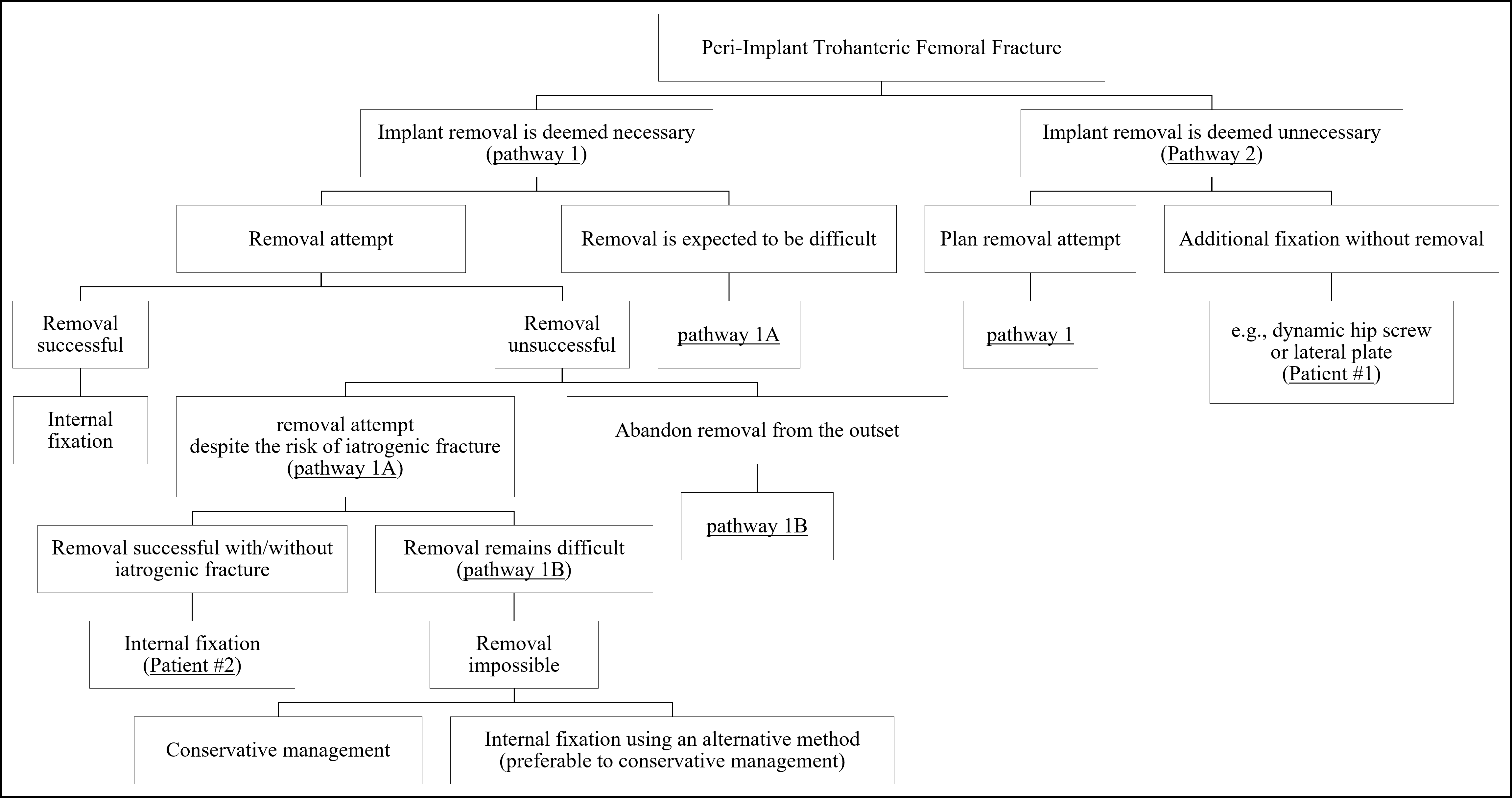

Supplement: Supplementary file 1 — Supporting Information Additional supporting information can be found online in the Supporting Information section. Figure S1: Decision‐making algorithm for the surgical management of peri‐implant trochanteric femoral fractures after intramedullary nailing. The algorithm is divided into two main pathways depending on whether implant removal is deemed necessary. Pathway 1 (removal necessary) includes detailed subbranches for removal attempts and the risk of iatrogenic fracture. Pathway 2 (removal not necessary) shows options for supplementary fixation around the retained implant. Patient #1 was managed according to Pathway 2 (additional fixation without removal using a dynamic hip screw), while Patient #2 followed Pathway 1 with successful nail extraction despite an iatrogenic fracture. [file CRO-2026-5170842-s001.tif]
